# Serial changes in the proliferation and differentiation of adipose-derived stem cells after ionizing radiation

**DOI:** 10.1186/s13287-016-0378-0

**Published:** 2016-08-17

**Authors:** Woonhyeok Jeong, Xiao Yang, Jeongmi Lee, Youngwook Ryoo, Jinhee Kim, Youngkee Oh, Sunyoung Kwon, Dalie Liu, Daegu Son

**Affiliations:** 1 Department of Plastic and Reconstructive Surgery, Institute for Medical Science, Keimyung University School of Medicine, Daegu, Republic of Korea; 2 Department of Plastic and Reconstructive Surgery, Zhujiang Hospital, Southern Medical University, Guangzhou, China; 3 Department of Dermatology, Institute for Medical Science, Keimyung University School of Medicine, Daegu, Republic of Korea; 4 Department of Radiation Oncology, Keimyung University School of Medicine, Daegu, Republic of Korea; 5 Department of Pathology, Keimyung University School of Medicine, Daegu, Republic of Korea

**Keywords:** Mesenchymal stromal cells, Radiation, Senescence, Cell differentiation, Cell proliferation, Swine

## Abstract

**Background:**

Adipose-derived stem cells (ASCs) are important to homeostasis and the regeneration of subcutaneous fat. Hence, we examined the proliferation and differentiation capacity of irradiated ASCs over time.

**Methods:**

Two female pigs received a single 18 Gy dose of ionizing radiation to an 18 × 8 cm area on the dorsal body skin via a 6 MeV electron beam. After irradiation, the ASCs were cultured from adipose tissue harvested from a non-irradiated area and an irradiated area at 2, 4, and 6 weeks. The proliferation capacity of ASCs was evaluated by a colony-forming units–fibroblasts (CFUs-Fs) assay, a cholecystokinin (CCK) test with 10 % fetal bovine serum (FBS), and a 1 % FBS culture test. The senescence of ASCs was evaluated through morphological examination, immunophenotyping, and β-galactosidase activity, and the multipotent differentiation potential of ASCs was evaluated in adipogenic, osteogenic, and chondrogenic differentiation media.

**Results:**

Irradiated ASCs demonstrated significantly decreased proliferative capacity 6 weeks after irradiation. As well, the cells underwent senescence, which was confirmed by blunted morphology, weak mesenchymal cell surface marker expression, and elevated β-galactosidase activity. Irradiated ASCs also exhibited significant losses in the capacity for adipocyte and chondrocyte differentiation. In contrast, osteogenic differentiation was preserved in irradiated ASCs.

**Conclusions:**

We observed decreased proliferation and senescence of irradiated ASCs compared to non-irradiated ASCs 6 weeks after irradiation. Furthermore, irradiated ASCs demonstrated impaired adipocyte and chondrocyte differentiation but retained their osteogenic differentiation capacity. Our results could shed light on additional pathogenic effects of late irradiation, including subcutaneous fibrosis and calcinosis.

## Background

Radiotherapy is an important treatment option for cancer patients, and approximately 60 % of cancer patients will receive radiotherapy during the course of their treatment [1]. Although radiotherapy is an effective treatment for cancer, ionizing radiation has side effects on the surrounding normal tissue, including skin and adipose tissue, and can cause injury to these tissues.

Subcutaneous fat is composed of various types of cells including adipocytes, mesenchymal stem cells termed as adipose tissue-derived stem cells (ASCs), vascular endothelial cells, and immune cells. Mesenchymal stem cells including bone marrow-derived mesenchymal stem cells (BMSCs) and ASCs have various functions as progenitor cells and differentiate into adipocytes, osteoblasts, and chondrocytes [2, 3]. ASCs also secrete many growth factors and cytokines, and improve wound healing by paracrine effects [4]. Injury to adipose tissue can result in replacement with fibrotic tissue or regeneration of adipose tissue. Further, patients that receive radiotherapy frequently experience atrophy of subcutaneous tissue and fibrosis; however, the mechanisms by which the subcutaneous tissue atrophy and fibrosis occur remain unclear. The findings of a previous investigation indicated that BMSCs are influenced by irradiation and lose their osteogenic differentiation potential as a result of DNA damage and senescence [5]. However, the influence of irradiation on ASCs has not yet been investigated and remains unclear. Therefore, we questioned whether the influence of radiation on ASCs might be related to subcutaneous atrophy and heterotrophic calcification.

Cutaneous radiation injuries were categorized as acute radiation injuries and delayed effects of irradiation. Traditionally, delayed effects of radiation injury are explained by decreased microcirculation with small artery and capillary occlusions [6]. As well, decreased microcirculation induces delayed wound healing and fibrosis. However, decreased microcirculation is unable to fully account for subcutaneous fat atrophy without trauma or wound development in patients that received irradiation. Furthermore, the calcification of subcutaneous fat, termed subcutaneous calcinosis, rarely developed in irradiated patients with unclear pathophysiology. Previous results indicated that ASCs were important to subcutaneous fat regeneration and homeostasis [7, 8]. Therefore, we suspected that chronological changes in ASCs might be closely related to delayed effects of irradiation in irradiated individuals.

## Methods

### Irradiation and harvesting of porcine adipose tissue

Two female micro pigs (Micropig®; Medikinetics, Pyeongtaek, Korea), older than 7 months of age, weighing 30 to 32 kg, and with no apparent skin diseases, were used. The experiment was performed in duplicate to obtain two replicates for each of the pigs. At 6 months of age, the micro pigs had completed the development of secondary sexual characteristics and were sufficiently mature. The pigs were fed a restricted feed during the experimental period, which controlled their growth and permitted the convenient examination of the wound contraction process.

One week prior to the experiment, the pigs were moved from the breeding farm and transported to the laboratory to allow for acclimation. Each pig was housed in a separate cage and was given 400 g of standardized gamma-irradiated feed and 3 liters of water per day. The laboratory was maintained at 21–23 °C with a relative humidity of 53–59 %. The Keimyung University School of Medicine Institutional Animal Care and Use Committee approved all experimental procedures involving the animals.

**Fig. 1:**
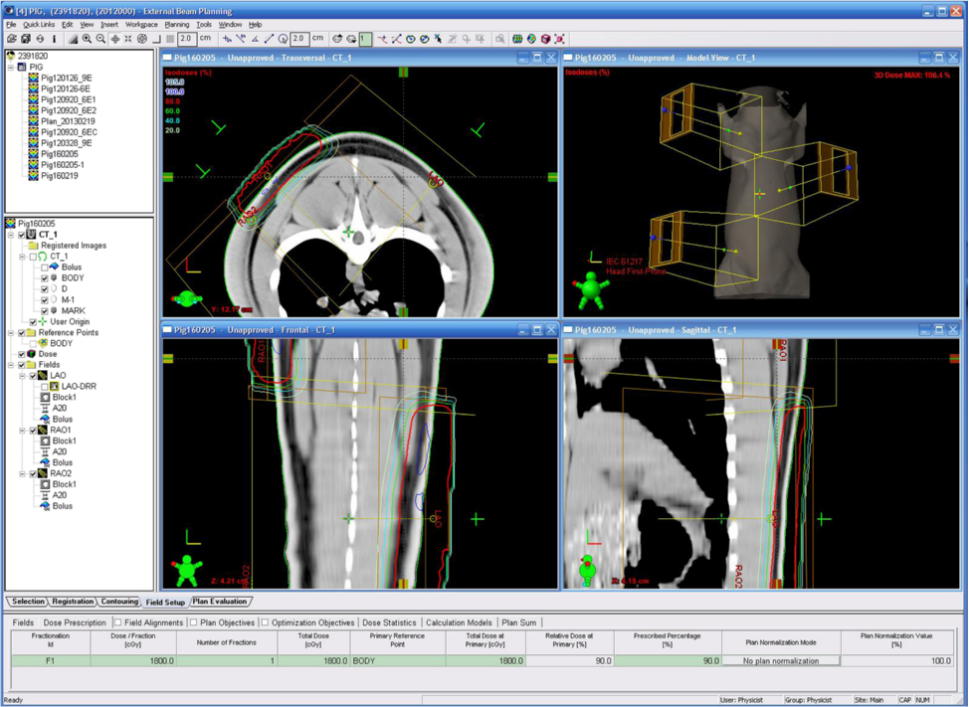
The simulation of irradiation level using simulation software. The 18 Gy dose of radiation level is delineated by the *red line*

On the day radiation was delivered, the pigs were anesthetized with tiletamine-zolazepam (Zoletil®; Virbac Laboratories, Carros, France) and xylazine hydrochloride (Rompun®; Bayer, Leverkusen, Germany). Before irradiation, skin thickness was measured by computed tomography (SOMATOM Sensation 16; Siemens AG, Forchheim, Germany) to simulate the radiation level using simulation software (Eclipse™ treatment planning system; Varian Medical Systems, Palo Alto, CA, USA; Fig. 1). Three areas on the paraspinal dorsal skin surface of each pig, two on the left side of the spine and one on the right side of the spine, were selected. The purpose of this design was to ensure that there was enough non-radiated tissue around each wound to avoid skin necrosis due to large-area radiation. Each pig received a single 18 Gy dose of radiation to an 18 × 8 cm area with a 6 MeV electron beam using a linear accelerator (Rapidarc®; Varian Medical Systems, Palo Alto, CA, USA). The radiation level was calculated to ensure that more than 90 % of the prescribed dose would be limited to a maximum depth of 2 cm. The borders of the irradiated fields were delineated to confirm the precise treatment of the area. Afterward, the animals were transported to the animal laboratory and housed under standard conditions.

### Preparation of n-ASCs and r-ASCs

#### Isolation and culturing of autologous and normal adipose-derived stem cells (n-ASCs)

Adipose tissues were harvested from a 6 × 4 cm paraspinal cutaneous flap that was made in the non-irradiated dorsal area. After harvesting the adipose tissues, the flap was closed with 1-0 nylon suture, and the adipose tissue samples were trimmed and transferred to sterile 50-ml conical tubes containing 25 ml phosphate-buffered saline (PBS). The fat tissues were washed twice with PBS and minced using a No. 10 blade. The total volume of the minced fat tissue was approximately 40 ml for each pig; the minced fat tissue was subsequently digested with 0.075 % collagenase type I (Worthington Biochemical Corporation, Lakewood, NJ, USA) in PBS at 37 °C for 1 hour under constant, moderate agitation. Afterward, culture medium containing high-glucose Dulbecco’s modified Eagle medium (DMEM) and 10 % fetal bovine serum (FBS) was added to halt the enzymatic activity. After centrifugation, the supernatant was discarded and the pellet was resuspended and filtered through a 100-μm cell strainer to remove tissue debris. The suspension was centrifuged again at 1500 rpm for 5 min and resuspended in low-glucose DMEM with 10 % FBS, seeded into 100Ø culture dishes, and incubated at 37 °C with 5 % CO_2_. The medium was then changed and the first-passage cells were frozen. According to an established schedule, the cells were thawed and cultured. Cells from the third passage were used for cell assays and cellular wound therapy. Similarly, n-ASCs used for the cell assays were harvested from adipose tissue excised from normal wounds created to serve as a negative control group.

#### Isolation and culture of radiation-injured adipose-derived stem cells (r-ASCs)

In total, three wounds were generated per pig. Every time a wound was created, the fat tissue from the radiation-injured zone was harvested, trimmed, and transferred to sterile 50-ml conical tubes containing 25 ml PBS after careful removal of the skin tissue, including the dermis. The fat tissue was then washed, minced, digested, and cultured as described for n-ASCs. When the number of r-ASCs obtained was sufficient, the cells were used in assays to compare with n-ASCs harvested from the normal wound group. According to the wound creation time, there were three groups of r-ASCs: (1) r-ASCs at 2 weeks post-radiation (2R group); (2) r-ASCs at 4 weeks post-radiation (4R group); (3) r-ASCs at 6 weeks post-radiation (6R group). Similarly, there were three groups of n-ASCs: (1) n-ASCs at 2 weeks post-radiation (2 N group); (2) n-ASCs at 4 weeks post-radiation (4 N group); (3) n-ASCs at 6 weeks post-radiation (6 N group).

### Evaluation of adipose-derived stem cells

#### Cell proliferation assay

r-ASCs and n-ASCs obtained following wound generation were seeded at a density of 1 × 10^4^ cells/well in DMEM with 1 % FBS and 10 % FBS, respectively. The N group indicates a mixed cell population comprised of the 2 N, 4 N, and 6 N groups. The growth rates of the cells were determined using the Cell Counting Kit-8 assay (CCK-8 assay; Dojindo Laboratories, Kumamoto, Japan). The media was changed every 3 days and the CCK-8 working solution was added at 3-day intervals up to day 11, followed by incubation of the cells for 2 h at 37 °C. The absorbance was measured at 450 nm using a microplate spectrophotometer. The numbers of cells were counted in the Automated Cell Counter (Luna-II®; Logos Biosystems, Anyang, Korea).

#### Senescence-associated β-galactosidase assay

r-ASCs and n-ASCs were plated in 24-well culture plates (1 × 10^4^ cells/well). Twenty-four hours later, the cells were washed with PBS and fixed in a fixative solution for 15 min, followed by three washes in PBS, and staining using the Senescence β-Galactosidase staining kit (Cell Signaling, Danvers, MA, USA). After incubation at 37 °C overnight, positively stained cells were counted by light microscopy under ×100 magnification.

#### Colony-forming units–fibroblast assay

r-ASCs and n-ASCs were resuspended and plated at a density of 1 × 10^2^ ~ 10^3^ cells in triplicate. Non-adherent cells were removed during a media change twice weekly. On day 15, the cells were fixed with 4 % paraformaldehyde for 10 minutes and stained with 0.5 % crystal violet (Millipore Sigma, St. Louis, MO, USA) in 10 % methanol for 20 minutes. For quantitative analysis, the colonies were resuspended in 100 % methanol for 5 minutes. Crystal violet absorbance was measured at 570 nm using a microplate spectrophotometer.

#### Immunophenotyping

Third-passage ASCs were harvested by treatment with a cell-dissociating enzyme (TrypLE™ Express; Thermo Fisher Scientific, Waltham, MA, USA) and washed twice with PBS. Cell aliquots (1 × 10^6^ cells/1 ml) were incubated for 30 min on ice with CD31 (BD Biosciences, San Jose, CA, USA), CD45 (AbD Serotec, Kidlington, UK), CD29 (BD Biosciences, San Jose, CA, USA), and CD90 (Abcam, Cambridge, UK) monoclonal antibodies. Isotype-matched normal mouse IgGs were used as controls (Abcam, Cambridge, UK). Flow cytometry was performed on a FACSCanto™II flow cytometer (BD Biosciences, San Jose, CA, USA) and data analysis was performed using FACSDiva™ version 6.1.3 (BD Biosciences, San Jose, CA, USA).

#### Reverse transcription-polymerase chain reaction

Total RNA was extracted from normal and irradiated cells according to a previously published protocol [9]. The RNA pellets were eluted in RNase-free water and stored at −80 °C until analysis. Each RNA sample (2 μg) was reverse transcribed to obtain cDNA using the PrimeScript™ RT reagent kit (Takara Bio Inc., Shiga, Japan) according to the manufacturer’s instructions. The resulting cDNA was diluted in a 1∶5 ratio with water and stored at −20 °C. To evaluate the transcription levels of the different genes, real-time PCR was performed using a LightCycler® 96 System (Roche Diagnostics, Basel, Switzerland) with SYBR® Premix Ex Taq™ (Takara Bio Inc., Shiga, Japan) and specific primers. Each sample was measured in triplicate using the following conditions: 10 min at 95 °C followed by 40 amplification cycles (5 s at 95 °C and 30 s at 60 °C) and a dissociation cycle (5 s at 95 °C, 1 min 60 °C, and 30 s at 95 °C). The expression of individual genes was normalized relative to the expression of glyceraldehyde 3-phosphate dehydrogenase (GAPDH), and the expression levels were calculated using the 2^ΔCt^ method, where ΔCt was determined by subtracting the GAPDH value from the target Ct. The following primers were used to amplify the specific endogenous mRNAs: PPAR-γ forward, 5′-GCG CCC TGG CAA AGC ACT-3′ and reverse, 5′-TCC ACG GAG CGA AAC TGA-3′; aP2 forward, 5′-GGC CAA ACC CAA CCT GA-3′ and reverse, 5′-GGG CGC CTC CAT CTA AG-3′; type II collagen forward, 5′-CCG GGC AGA GGG CAA TAG CAG GTT-3′ and reverse, 5′-CAA TGA TGG GGA GGC GTG AG-3′; aggrecan forward, 5′-CCA GAA TCT AGC AGG GAG TCA TC-3′ and reverse, 5′-AGG CAG AGG TGG CTT CAG TC-3′; type I collagen forward, 5′-CCA AGA GGA GGG CCA AGA AGA AGG-3′ and reverse, 5′-GGG GCA GAC GGG GCA GCA CTC-3′; osteocalcin forward, 5′-TCA ACC CCG ACT GCG ACG AG-3′ and reverse, 5′-TTG GAG CAG CTG GGA TGA TGG-3′.

#### Differentiation assay

The ASCs were incubated with standard adipogenic (Zen-Bio, Inc., Research Triangle Park, NC, USA), osteogenic (PromoCell, Heidelberg, Germany), or chondrogenic differentiation medium (PromoCell, Heidelberg, Germany). To quantify the adipogenic potential, the cultures were stained with Oil Red O (Millipore Sigma, St. Louis, MO, USA) to elucidate lipid droplets. To quantify the osteogenic potential, cultures were fixed with 10 % formaldehyde and stained with Alizarin Red S (Millipore Sigma, St. Louis, MO, USA) that was solubilized with 10 % acetic acid neutralized with 10 % ammonium hydroxide. Alkaline phosphatase activity (AP activity; AnaSpec, Fremont, CA, USA) was detected by p-nitrophenyl phosphate (pNPP). To assess the chondrogenic potential, the cultures were stained with hematoxylin and eosin (H&E) and Alcian blue, followed by immunohistochemistry with collagen type II (Bioss Antibodies, Woburn, MA, USA).

#### Sulfated glycosaminoglycan (sGAG) assay

Pellet cultures were digested overnight at 60 °C with 300 μg/ml papain (Millipore Sigma, St. Louis, MO, USA) in 20 mM sodium phosphate buffer (pH 6.8) containing 5 mM EDTA and 2 mM dithiothreitol (DTT). Cell lysates were clarified by centrifugation and sGAG was determined using the Blyscan™ sGAG assay kit (Biocolor Ltd, Carrickfergus, UK) according to the manufacturer’s protocol. Briefly, cell lysates were incubated with the 1,9-dimethylmethylene blue (DMMB) dye reagent for 30 min and unbound dye was removed by centrifugation. The bound dye was dissociated from the sGAG–dye complex and quantified by spectrophotometry based on A656. Using chondroitin 4-sulfate as a standard, total sGAG was determined and expressed as a function of the protein content.

#### ELISA for the quantification of leptin

The leptin concentration in the culture medium was determined using a sandwich ELISA and normalized to the protein concentration. The cell culture medium was removed on day 20 and centrifuged for 5 min at 12,000 rpm to remove the cellular debris, after which the supernatant was frozen at −80 °C prior to use. The leptin concentration was assessed using the Porcine Leptin Enzyme-Linked Immunosorbent Assay kit (Uscn Life Science Inc., Wuhan, China). The protein concentration was determined using the Pierce BCA® Protein Assay kit (Thermo Fisher Scientific, Waltham, MA, USA).

### Statistical analysis

The results were analyzed by the Kruskal-Wallis test with Dunn’s post hoc test using GraphPad Prism 5® (GraphPad Software Inc., San Diego, CA, USA) and are presented as the mean ± SEM. Values of *p* < 0.05 were considered statistically significant.

## Results

### Irradiation inhibits the proliferation of ASCs following a latency period of 6 weeks

**Fig. 2:**
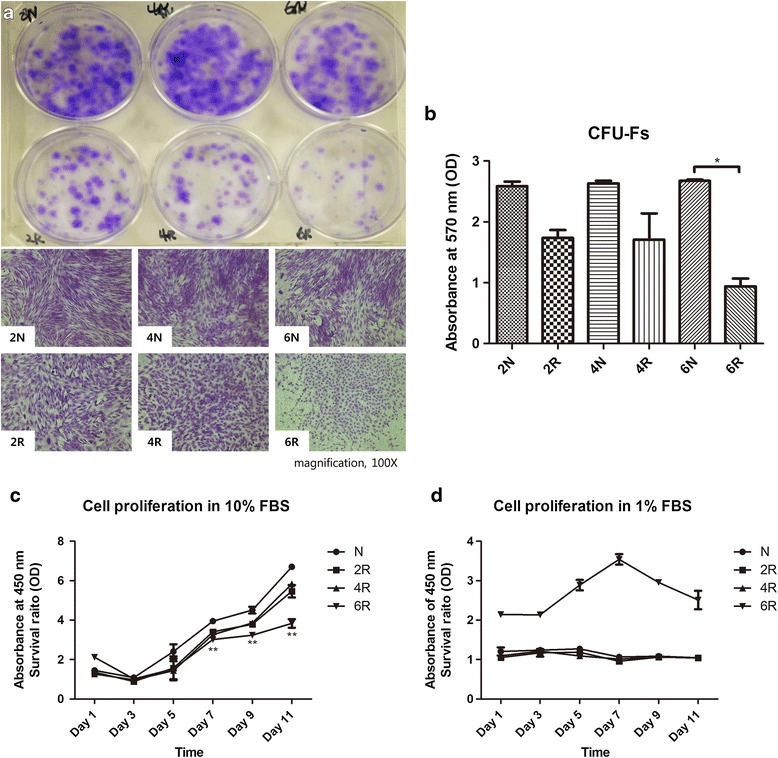
Proliferation capacity. **a** Macroscopic and microscopic view of colony-forming units (CFUs) determined by crystal violet staining. The number of viable ASC colonies formed was significantly more abundant in the N groups than in the 6R group. **b** Quantitative analysis of CFUs. CFU formation in the 6 N group was 2.5-fold higher than in the 6R group (^*^ *p* < 0.05). **c** Cell Counting Kit-8 (CCK-8) assay with 10 % fetal bovine serum (FBS). The cell numbers in the 6R group were significantly lower than numbers in the N group after day 7 (^**^ *p* < 0.01). **d** CCK-8 assay with 1 % FBS to analyze cellular growth under stress conditions. The cell numbers in the 6R group were higher than in the other groups throughout the entire experimental period. However, statistically significant differences were not obtained

The formation of viable ASC colonies was significantly higher in the N groups than in the 6R group (Fig. 2a). The absorbance of crystal violet at 570 nm was also significantly decreased in the 6R group compared to the 6 N group (^*^p < 0.05; 2 N group, 2.59 ± 0.07 OD; 2R group, 1.74 ± 0.13 OD; 4 N group, 2.63 ± 0.04 OD; 4R group, 1.71 ± 0.43 OD; 6 N group, 2.68 ± 0.02 OD; 6R group, 0.94 ± 0.13 OD; Fig. 2b). Although the numbers of viable ASCs in the 2R and 4R groups were lower than in the 2 N and 4 N groups, the difference in crystal violet absorbance was not statistically significant.

The rests of the proliferation test, as determined by the CCK-8 assay, indicated that all groups demonstrated serial cell growth with 10 % FBS. However, the 6R group demonstrated a plateau in cell growth and less cellular proliferation than the N group after day 7 (^**^p < 0.01; Fig. 2c). Meanwhile, the proliferation rate of ASCs was higher in the 6R group than the rates in the other groups when medium containing 1 % FBS was used as a cell stress test. However, none of the differences between the groups were statistically significant (Fig. 2d).

### Irradiation induces senescence of ASCs following a latency period of 6 weeks

**Fig. 3:**
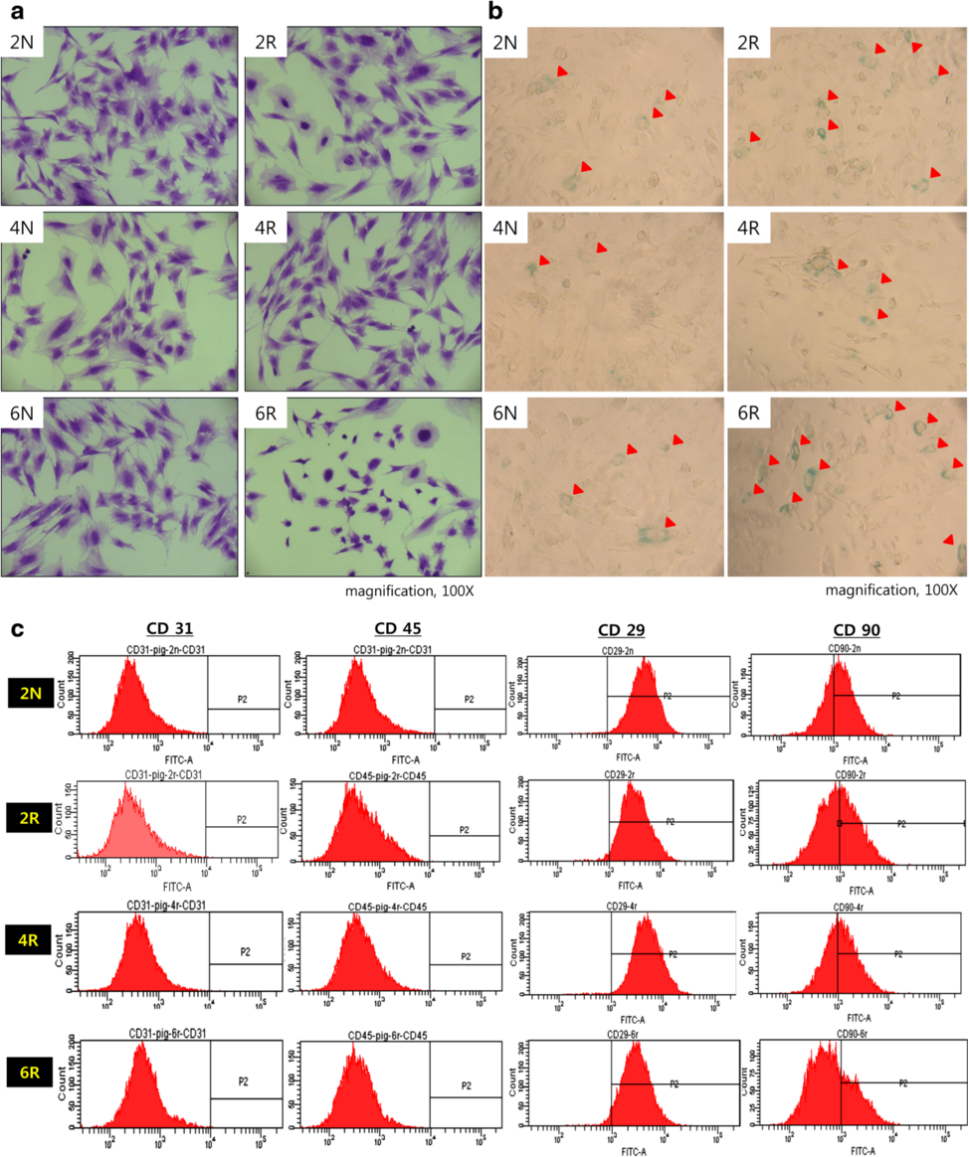
Senescence of irradiated ASCs. **a** Crystal violet staining. Cell morphology was inhomogeneous, smaller, and blunter in the 6R group compared to the other groups. **b** β-galactosidase immunostaining. Positive β-galactosidase staining (*arrowhead*) was rarely observed in the N groups, but was readily apparent in the 6R group. **c** Immunophenotyping by flow cytometry. All groups demonstrated reduced expression of the hematopoietic surface markers CD 31 and CD41. The 6R groups demonstrated lower CD90 expression than the other groups

After crystal violet staining, the morphology of the cells in the 6R group was inhomogeneous, smaller, and blunt compared to the other groups (Fig. 3a). Positive immunostaining for senescence-associated β-galactosidase was rarely observed in the N groups but was readily apparent in the 6R group (Fig. 3b). Flow cytometry was performed to characterize the phenotypes of irradiated and non-irradiated ASCs. We presented the 2 N group as the control group in Fig. 3c because all of the normal groups demonstrated similar surface marker expression. The expression of CD45 and CD31, the hematopoietic and endothelial lineage markers, respectively, was not detected in all of the groups. The N group demonstrated increased expression of the mesenchymal origin cell surface markers CD29 and CD90, identical to the immunophenotype of normal ASCs. However, irradiated ASCs demonstrated decreased expression of CD90, particularly in the 6R group (Fig. 3c).

### Irradiated ASCs lose the capacity for adipogenic and chondrogenic differentiation but retain the capacity for osteogenic differentiation

**Fig. 4:**
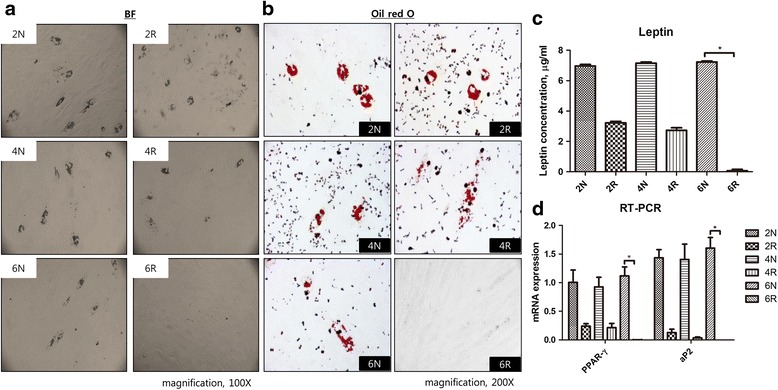
Adipogenic differentiation. **a** Bright field view. Adipogenic differentiation was not observed in the 6R group. **b** Oil Red O staining. Loss of adipogenic differentiation in the 6R group was confirmed by Oil Red O staining. **c** Leptin analysis. The secretion of leptin hormone from adipocytes was significantly lower in the 6R group than in the 6 N group (^*^ *p* < 0.05). **d** RT-PCR for PPAR-γ and aP2. The levels of PPAR-γ and aP2 mRNA were significantly lower in the 6R group than in the 6 N group (^*^ *p* < 0.05)

After culturing in adipogenic induction media, adipogenic differentiation was measured by bright field and Oil Red O staining at day 20. The differentiation of adipocytes was lacking in the 6R group, whereas the other groups demonstrated adipogenic differentiation (Fig. 4a). The loss of adipogenic differentiation in the 6R group was confirmed by Oil Red O staining (Fig. 4b). The lipid content was determined by an ELISA that measured leptin, a hormone primarily produced by fat cells. The amount of leptin was significantly reduced in the 6R group compared with the 6 N groups (*p* < 0.05; 2 N group, 6.96 ± 0.11 μg/mL; 2R group, 3.22 ± 0.09 μg/mL; 4 N group, 7.15 ± 0.08 μg/mL; 4R group, 2.73 ± 0.17 μg/mL; 6 N group, 7.23 ± 0.06 μg/mL; 6R group, 0.07 ± 0.09 μg/mL; Fig. 4c). Further, the relative mRNA expression of PPAR-γ, a key adipogenic transcription factor, was significantly suppressed in the 6R group compared with the 6 N group (*p* < 0.05; 2 N group, 1.01 ± 0.21; 2R group, 0.24 ± 0.04; 4 N group, 0.93 ± 0.17; 4R group, 0.21 ± 0.07; 6 N group, 1.12 ± 0.17; 6R group, 0.002 ± 0.00; Fig. 4d). The aP2 gene, which encodes an adipocyte-specific protein, was also significantly suppressed in the 6R group compared with the N group (*p* < 0.05; 2 N group, 1.44 ± 0.14; 2R group, 0.13 ± 0.06; 4 N group, 1.41 ± 0.27; 4R group, 0.04 ± 0.02; 6 N group, 1.60 ± 0.18; 6R group, 0.00 ± 0.00; Fig. 4d).

**Fig. 5:**
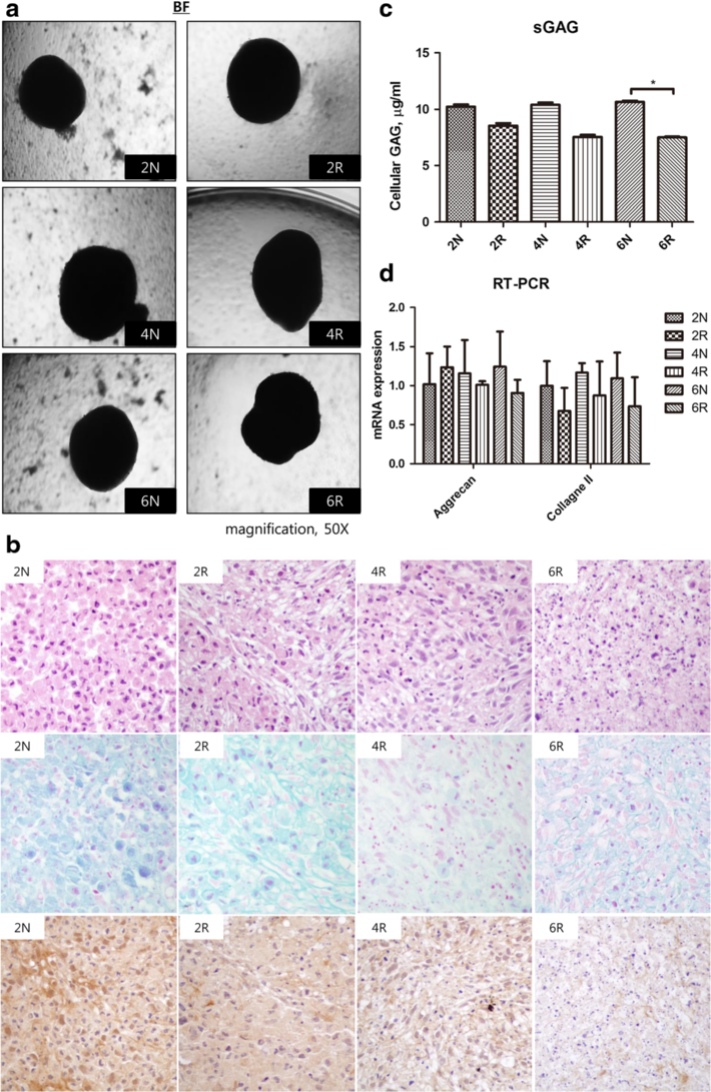
Chondrogenic differentiation. **a** Bright field view. All of the groups exhibited chondrogenic differentiation. **b** H&E staining (*first row*) demonstrated that cultured cartilage cells are prominently increased in the 2 N group in the absence of necrosis or apoptosis. However, cartilage cells are decreased and substituted for necrotic cells and apoptosis in the 4R and 6R groups. Alcian blue staining (*second row*) likewise demonstrated that cultured cartilage cells declined remarkably as the weeks after exposure to radiation progressed. Immunohistochemistry for type II collagen (*third row*) demonstrated positivity for cytoplasmic localization of the cultured cartilage cells in the 2 N group. Viable cartilage cells are markedly reduced and replaced with necrotic cells with negative collagen type II antibody expression in the 4R and 6R groups. **c** Sulfated glycosaminoglycan (sGAG) assay. The level of sGAG was significantly lower in the 6R group than in the 6 N group (^*^ *p* < 0.05). **d** RT-PCR for aggrecan and type II collagen did not reveal any statistically significant differences

To determine chondrogenic differentiation, the cultures were stained with H&E and Alcian blue, followed by immunohistochemistry with type II collagen on day 21. All of the groups exhibited chondrogenic differentiation in bright field view (Fig. 5a). Based on H&E staining, the number of cultured cartilage cells increased in the 2 N group, and necrosis and apoptosis were absent. However, the number of cells that differentiated from irradiated ASCs decreased and necrosis and apoptosis were observed, particularly in the 4R and 6R groups (Fig. 5b, first row). The results of Alcian blue staining likewise demonstrated that differentiated cartilage cells declined in the 4R and 6R groups (Fig. 5b, second row). Immunohistochemistry of type II collagen revealed the cytoplasmic localization of differentiated cartilage cells in the 2 N group, whereas the 4R and 6R irradiated groups demonstrated significantly weak staining (Fig. 5b, third row). The concentration of sulfated glycosaminoglycan (sGAG), a component of cartilage that is situated on aggrecan, was quantified, and the results revealed that sGAGs were significantly reduced in the 6R group compared with the 6 N group (*p* < 0.05; 2 N group, 10.23 ± 0.21 μg/mL; 2R group, 8.54 ± 0.23 μg/mL; 4 N group, 10.41 ± 0.20 μg/mL; 4R group, 7.54 ± 0.19 μg/mL; 6 N group, 10.65 ± 0.12 μg/mL; 6R group, 7.50 ± 0.07 μg/mL; Fig. 5c). However, we did not detect significant differences in the mRNA levels of aggrecan or type II collagen, the major structural components of cartilage (Fig. 5d). In summary, the results indicated that the chondrogenic differentiation of ASCs was impaired 6 weeks after irradiation.

**Fig. 6:**
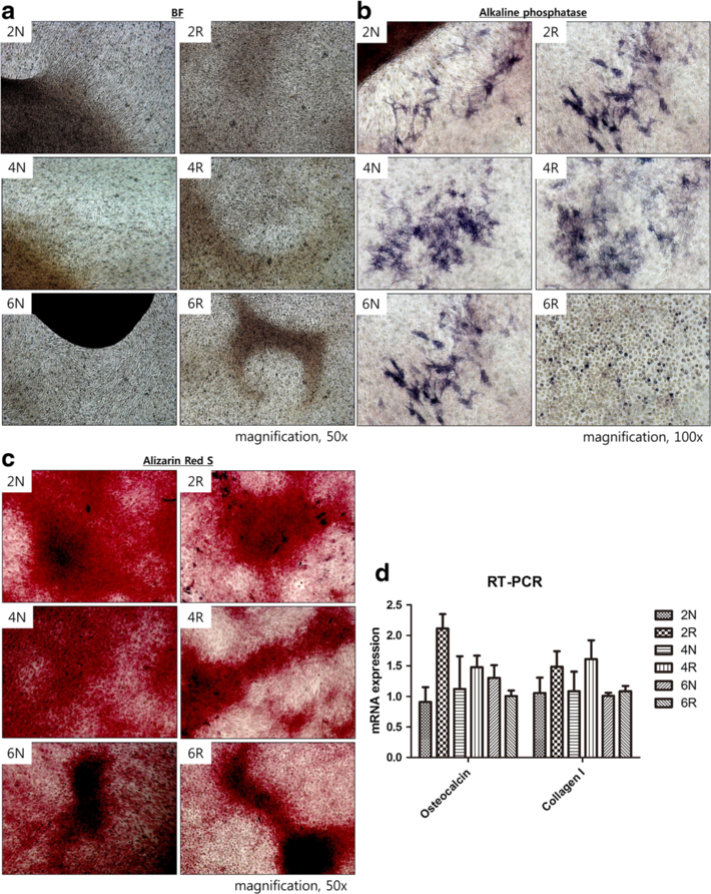
Osteogenic differentiation. **a** Bright field view. Calcium deposition (*black dots*) was scattered throughout the entire area after osteogenic differentiation in all groups. Calcium deposition was condensed and became a mineralized spot (*brown and black area*) in all groups. **b** Alkaline phosphatase (AP) activity. All of the groups demonstrated AP activity. Although the 6R group demonstrated the small and round shape morphology of senescent cells, AP activity was also detected in the senescent cells. **c** Alizarin Red S staining. Mineralization of osteogenically differentiated ASCs was confirmed in all groups by Alizarin Red S staining. **d** RT-PCR for osteocalcin and type I collagen. No statistically significant differences in osteocalcin type I collagen mRNA levels were detected between the groups

After osteogenic induction, ASCs in all of the groups differentiated into osteoblasts by day 18. Using bright field microscopy, mineralization was identified in all of the groups (Fig. 6a). AP activity was used as an early marker of osteogenic differentiation and was detected in all groups. All control groups, as well as the 2R and 4R groups, demonstrated strong AP activity. Although the 6R group exhibited the small and round shape morphology of senescent cells, AP activity was also detected (Fig. 6b). Alizarin Red S staining was performed to confirm the mineralization associated with osteogenic differentiation and the results indicated that all of the groups demonstrated osteogenic differentiation with abundant mineralization (Fig. 6c). Although the expression of the gene that encodes osteocalcin, a noncollagenous protein found in bone, was most profound in the 2R group, the differences between the groups were not statistically significant. As well, the expression of the type I collagen gene, which is the major collagen in bone, was not statistically different between the groups (Fig. 6d), which indicated that the osteogenic differentiation of ASCs was not suppressed by irradiation.

## Discussion

In this study, we examined chronologic changes in irradiated ASCs using proliferation and differentiation assays. We discovered that the proliferation of ASCs was impaired, with senescence 6 weeks after irradiation compared with controls and shorter post-irradiation time points. Further, ASCs with impaired proliferation and senescence exhibited less adipogenic and chondrogenic differentiation in contrast to non-irradiated ASCs, but did not exhibit impaired osteogenic differentiation.

Traditionally, the delayed effects of irradiation injury have been explained by decreased microcirculation accompanied by small artery and capillary occlusion [6]. However, atrophy of subcutaneous fat or wound development could not be adequately explained only by decreased microcirculation. It has been established in previous investigations that ASCs are important to the homeostasis of subcutaneous fat [7, 10, 11]. Therefore, we hypothesized that the chronological changes in ASCs might be closely related to the delayed effects of irradiation in individuals that have undergone irradiation. However, previous studies that have investigated the response of mesenchymal stem cells (MSCs) to irradiation were conducted in vitro, and thus could only examine the immediate effects of irradiation [5, 12–16]. In this study, irradiation was performed in vivo and the ASCs were serially harvested from live animals. Therefore, we could investigate the delayed effects of irradiation over a period of time in contrast to previous investigations that were performed in vitro.

Ionizing radiation leads to DNA damage by direct deposition of energy in the bases and phosphate backbone of DNA, or by indirectly ionizing water molecules to produce radical superoxides that damage DNA [17]. DNA repair mechanisms are initiated after irradiation and the possible fates of cells include DNA repair, cell cycle arrest, senescence, and apoptosis, depending on the severity of the DNA damage [18]. In a prior study, MSCs that possessed radio-resistance underwent senescence rather than apoptosis following high-dose irradiation (20 Gy) [14]. Our results on the proliferation of irradiated ASCs indicate that the colony-forming units were significantly decreased in the 6R group compared to the other groups. Cellular growth as determined by the CCK-8 assay with 10 % FBS was also significantly decreased after day 7 in the 6R group. Interestingly, although we did not detect statistically significant differences, the proliferation capacity of the 6R group was less influenced by the 1 % FBS stress condition than other groups, which could be explained by the fact that these cells had already survived in the harsh conditions induced by irradiation. Hence, it appears that impaired proliferation had a latency period of 6 weeks, which we suspect was caused by an increase in the number of senescent ASCs accumulated with the passage of time.

Senescence is a condition of permanent cell cycle arrest that is characterized by decreased proliferation, morphological changes, and increases in senescence-associated β-galactosidase activity [12]. Senescent cells exhibit morphological characteristics that include flattening and enlarged cytoplasm with increased granularity [18]. Six weeks after irradiation, the ASCs exhibited a smaller, blunt shape and easily detached from the culture plate. β-galactosidase activity, which is an established marker of senescent cells, was also markedly increased compared to the other groups [18]. The proportion of senescent ASCs that had dis-morphogenesis and increased β-galactosidase activity was increased in the 6R group. As well, an increased number of senescent ASCs could explain this decreased proliferation capacity because the senescent cells demonstrated permanent cell cycle arrest [12]. Furthermore, CD90 was markedly decreased in the 6R group, whereas no differences in the hematopoietic surface markers CD31 and CD45 were detected. In prior studies, MSCs were identified by morphological characteristics, including fibroblast-like spindle cells and expression of the mesenchymal origin cell surface markers CD13, CD29, CD44, CD73, CD79, CD 105, and CD106 in the absence of the hematopoietic cellular markers CD31 and CD45 [19, 20]. In this regard, the senescent ASCs demonstrated loss of their ‘stemness’ with dis-morphogenesis and diminished expression of mesenchymal origin cell surface markers 6 weeks after irradiation.

ASCs are important to the homeostasis and repair of tissues in which they are found. Additionally, ASCs possess various functions as progenitor cells and differentiate into adipocytes, osteoblasts, and chondrocytes [3]. Injury to adipose tissue could result in fibrosis and the appearance of fibrotic tissue, or in the regeneration of adipose tissue. During the repair of adipose tissue, ASCs participate in the regeneration of adipocytes and in the suppression of fibroplasia [7]. Furthermore, ASCs play a crucial role in regenerating adipocytes after fat grafting [10, 11]. In our study, the results of Oil Red O staining indicated that adipogenic differentiation was significantly suppressed and that leptin levels were diminished in the 6R group. Leptin is a hormone secreted by adipocytes; it is concomitantly increased throughout adipocyte differentiation [21]. Hence, we confirmed a decrease in adipogenic differentiation in the 6R group by measuring leptin levels.

PPAR-γ is a member of the nuclear receptor superfamily and is a major transcription factor of adipogenesis [22, 23]. PPAR-γ appears prior to the activation of many other adipocyte genes during adipogenic differentiation and induces stem cells to differentiate into adipocytes [24]. The protein aP2 is highly expressed in mature adipocytes and was originally identified as an adipocyte-specific protein [25]. aP2 plays important roles in intracellular fatty acid transport and metabolism, especially in the maintenance of glucose levels and lipid homeostasis [26]. In our study, irradiation inhibited PPAR-γ, which was measured as a marker for adipocyte differentiation and adipogenesis. As well, our finding was confirmed by the suppression of aP2.

Histology results revealed that chondrogenic differentiation was impaired 4 weeks after irradiation, and occurred earlier than adipogenic differentiation. Alcian blue staining, which is specific for highly sulfated proteoglycans in the cartilage matrix, was weakly apparent 4 weeks after irradiation [27]. Immunohistochemistry demonstrated a decrease in the expression of type II collagen 4 weeks after irradiation. Further, the abundance of sGAGs, the extracellular components of cartilage, was concomitantly decreased 2 weeks after irradiation. However, we did not observe any significant differences in aggrecan and type II collagen mRNA expression by RT-PCR. Although mRNAs were eventually translated to protein, mRNA levels did not coincide with protein levels [28]. In particular, miRNA repressed the translation of mRNA into protein via post-transcriptional inhibition [29], which could explain the high mRNA levels but corresponding low protein levels of aggrecan and type II collagen in irradiated ASCs.

Unlike adipogenic and chondrogenic differentiation, osteogenic differentiation was not inhibited by irradiation. The delayed effects of radiation on the skin that develop several months after irradiation include telangiectasia, atrophy, fibrosis, and necrosis [30]. A rare delayed effect of irradiation was subcutaneous calcinosis, which is the development of heterotrophic calcification in the absence of a systemic imbalance of extracellular calcium and phosphate concentrations [31]. Although previous investigations reported that subcutaneous calcification resulted from impaired microcirculation followed by a disruption in the extracellular equilibrium of calcium, the pathogenesis of subcutaneous calcinosis remains unclear [32]. In our study, the impairment of proliferation and adipocyte differentiation of irradiated ASCs could explain the fat atrophy and subcutaneous fibrosis seen as delayed effects of irradiation. Furthermore, the observation that ASCs retained the capacity for osteogenic differentiation could be a potential cause of subcutaneous calcinosis after irradiation.

## Conclusions

This was a pilot study to investigate the effects of late irradiation on ASCs. We observed decreased proliferation of ASCs, with senescence 6 weeks after irradiation compared to non-irradiated ASCs. Furthermore, irradiated ASCs demonstrated impaired adipogenic and chondrogenic differentiation, whereas osteogenic differentiation was preserved. Our results likely represent additional late pathogenic effects of irradiation, including subcutaneous fibrosis and subcutaneous calcinosis.

## Abbreviations

2 N group, n-ASCs at 2 weeks post-radiation; 2R group, r-ASCs at 2 weeks post-radiation; 4 N group, n-ASCs at 4 weeks post-radiation; 4R group, r-ASCs at 4 weeks post-radiation; 6 N group, n-ASCs at 6 weeks post-radiation; 6R group, r-ASCs at 6 weeks post-radiation; AP activity, alkaline phosphatase activity; ASCs, adipose-derived stem cells; BMSCs, bone marrow-derived mesenchymal stem cells; CCK, cholecystokinin; CCK-8 assay, Cell Counting Kit-8 assay; CFUs-Fs, colony-forming units–fibroblasts assay; DMEM, Dulbecco’s modified Eagle’s medium; DMMB, 1,9-dimethylmethylene blue; DTT, dithiothreitol; FBS, fetal bovine serum; GAPDH, glyceraldehyde 3-phosphate dehydrogenase; H&E, hematoxylin and eosin; MSCs, mesenchymal stem cells; N group, sum of 2 N, 4 N, and 6 N groups; n-ASCs, normal adipose-derived stem cells; PBS, phosphate-buffered saline; pNPP, p-nitrophenyl phosphate; r-ASCs, radiation-injured adipose-derived stem cells; sGAG, sulfated glycosaminoglycan

## References

[1] Robbins ME, Brunso-Bechtold JK, Peiffer AM, Tsien CI, Bailey JE, Marks LB (2012). Imaging radiation-induced normal tissue injury. Radiat Res..

[2] Djouad F, Jackson WM, Bobick BE, Janjanin S, Song Y, Huang GT (2010). Activin A expression regulates multipotency of mesenchymal progenitor cells. Stem Cell Res Ther..

[3] Guilak F, Lott KE, Awad HA, Cao Q, Hicok KC, Fermor B (2006). Clonal analysis of the differentiation potential of human adipose-derived adult stem cells. J Cell Physiol..

[4] Toyserkani NM, Christensen ML, Sheikh SP, Sorensen JA (2015). Adipose-derived stem cells: new treatment for wound healing?. Ann Plast Surg..

[5] Despars G, Carbonneau CL, Bardeau P, Coutu DL, Beausejour CM (2013). Loss of the osteogenic differentiation potential during senescence is limited to bone progenitor cells and is dependent on p53. PLoS One..

[6] Hopewell JW, Campling D, Calvo W, Reinhold HS, Wilkinson JH, Yeung TK (1986). Vascular irradiation damage: its cellular basis and likely consequences. Br J Cancer Suppl..

[7] Suga H, Eto H, Shigeura T, Inoue K, Aoi N, Kato H (2009). IFATS collection: Fibroblast growth factor-2-induced hepatocyte growth factor secretion by adipose-derived stromal cells inhibits postinjury fibrogenesis through a c-Jun N-terminal kinase-dependent mechanism. Stem Cells..

[8] Suga H, Eto H, Aoi N, Kato H, Araki J, Doi K (2010). Adipose tissue remodeling under ischemia: death of adipocytes and activation of stem/progenitor cells. Plast Reconstr Surg..

[9] Chomczynski P (1993). A reagent for the single-step simultaneous isolation of RNA, DNA and proteins from cell and tissue samples. Biotechniques.

[10] Kato H, Mineda K, Eto H, Doi K, Kuno S, Kinoshita K (2014). Degeneration, regeneration, and cicatrization after fat grafting: dynamic total tissue remodeling during the first 3 months. Plast Reconstr Surg.

[11] Eto H, Kato H, Suga H, Aoi N, Doi K, Kuno S (2012). The fate of adipocytes after nonvascularized fat grafting: evidence of early death and replacement of adipocytes. Plast Reconstr Surg..

[12] Cmielova J, Havelek R, Soukup T, Jiroutova A, Visek B, Suchanek J (2012). Gamma radiation induces senescence in human adult mesenchymal stem cells from bone marrow and periodontal ligaments. Int J Radiat Biol..

[13] Singh S, Kloss FR, Brunauer R, Schimke M, Jamnig A, Greiderer-Kleinlercher B (2012). Mesenchymal stem cells show radioresistance in vivo. J Cell Mol Med..

[14] Muthna D, Soukup T, Vavrova J, Mokry J, Cmielova J, Visek B (2010). Irradiation of adult human dental pulp stem cells provokes activation of p53, cell cycle arrest, and senescence but not apoptosis. Stem Cells Dev..

[15] Oliver L, Hue E, Sery Q, Lafargue A, Pecqueur C, Paris F (2013). Differentiation-related response to DNA breaks in human mesenchymal stem cells. Stem Cells..

[16] Mussano F, Lee KJ, Zuk P, Tran L, Cacalano NA, Jewett A (2010). Differential effect of ionizing radiation exposure on multipotent and differentiation-restricted bone marrow mesenchymal stem cells. J Cell Biochem..

[17] Mahaney BL, Meek K, Lees-Miller SP (2009). Repair of ionizing radiation-induced DNA double-strand breaks by non-homologous end-joining. Biochem J..

[18] Eriksson D, Stigbrand T (2010). Radiation-induced cell death mechanisms. Tumour Biol..

[19] Chen BY, Wang X, Chen LW, Luo ZJ (2012). Molecular targeting regulation of proliferation and differentiation of the bone marrow-derived mesenchymal stem cells or mesenchymal stromal cells. Curr Drug Targets..

[20] Tolar J, Le Blanc K, Keating A, Blazar BR (2010). Concise review: hitting the right spot with mesenchymal stromal cells. Stem Cells..

[21] MacDougald OA, Hwang CS, Fan H, Lane MD (1995). Regulated expression of the obese gene product (leptin) in white adipose tissue and 3 T3-L1 adipocytes. Proc Natl Acad Sci U S A..

[22] Lee H, Bae S, Kim K, Kim W, Chung SI, Yoon Y (2010). Beta-catenin mediates the anti-adipogenic effect of baicalin. Biochem Biophys Res Commun..

[23] Yadav S, Anbalagan M, Shi Y, Wang F, Wang H (2013). Arsenic inhibits the adipogenic differentiation of mesenchymal stem cells by down-regulating peroxisome proliferator-activated receptor gamma and CCAAT enhancer-binding proteins. Toxicol In Vitro..

[24] Li J, Wang Y, Li Y, Sun J, Zhao G (2014). The effect of combined regulation of the expression of peroxisome proliferator-activated receptor-gamma and calcitonin gene-related peptide on alcohol-induced adipogenic differentiation of bone marrow mesenchymal stem cells. Mol Cell Biochem..

[25] Spiegelman BM, Frank M, Green H (1983). Molecular cloning of mRNA from 3T3 adipocytes. Regulation of mRNA content for glycerophosphate dehydrogenase and other differentiation-dependent proteins during adipocyte development. J Biol Chem.

[26] Elmasri H, Karaaslan C, Teper Y, Ghelfi E, Weng M, Ince TA (2009). Fatty acid binding protein 4 is a target of VEGF and a regulator of cell proliferation in endothelial cells. Faseb j..

[27] Zuk PA, Zhu M, Ashjian P, De Ugarte DA, Huang JI, Mizuno H (2002). Human adipose tissue is a source of multipotent stem cells. Mol Biol Cell..

[28] Greenbaum D, Colangelo C, Williams K, Gerstein M (2003). Comparing protein abundance and mRNA expression levels on a genomic scale. Genome Biol..

[29] Jackson RJ, Hellen CU, Pestova TV (2010). The mechanism of eukaryotic translation initiation and principles of its regulation. Nat Rev Mol Cell Biol..

[30] Brush J, Lipnick SL, Phillips T, Sitko J, McDonald JT, McBride WH (2007). Molecular mechanisms of late normal tissue injury. Semin Radiat Oncol..

[31] Walsh JS, Fairley JA (1995). Calcifying disorders of the skin. J Am Acad Dermatol..

[32] Amin R, Hamilton-Wood C, Silver D (2002). Subcutaneous calcification following chest wall and breast irradiation: a late complication. Br J Radiol..

